# Pemetrexed-Induced Interstitial Pneumonitis: A Case Study and Literature Review

**DOI:** 10.14740/wjon845w

**Published:** 2014-12-03

**Authors:** Michael J. Waters, Shawgi Sukumaran, Chris S. Karapetis

**Affiliations:** a Royal Adelaide Hospital, Adelaide, South Australia 5000, Australia; b Department of Medical Oncology, Flinders Medical Centre, Bedford Park, Adelaide, South Australia 5042, Australia

**Keywords:** Chemotherapy, Interstitial pneumonitis, Pemetrexed, Lung toxicity, Non-small cell lung cancer, Treatment-related complication

## Abstract

Pemetrexed is a new-generation antifolate drug, now widely used in patients with non-small cell lung cancer (NSCLC). We report a case of pemetrexed-induced interstitial pneumonitis, and review the literature of eight previously reported cases. As pemetrexed is now a widely used chemotherapeutic agent, it is important to be aware of rare adverse events related to its administration.

## Introduction

Pemetrexed is a new-generation antifolate drug. Intracellularly, pemetrexed and its polyglutamate derivatives potently inhibit multiple tetrahydrofolate-cofactor-requiring enzyme pathways [1]. It has hence been referred to as a “multitargeted” antifolate [2].

Pemetrexed is currently widely used in subsets of patients with non-small cell lung cancer (NSCLC): as first-line combination chemotherapy with cisplatin in patients with non-squamous pathology [3], as second-line monotherapy following initial treatment with platinum-based chemotherapy [4], and more recently as maintenance chemotherapy in non-progressing patients following platinum-based first-line chemotherapy [5]. Pemetrexed is also used as combination chemotherapy with cisplatin for malignant pleural mesothelioma [6]. The phase III clinical trials which led to the approval of pemetrexed in patients with NSCLC did not report any significant pulmonary toxicity [3, 7]. However, since 2009, eight reported cases have linked pemetrexed with acute interstitial pneumonitis. As pemetrexed is now a widely used chemotherapeutic agent, it is important to be aware of rare adverse events related to its administration.

## Case Report

**Figure 1:**
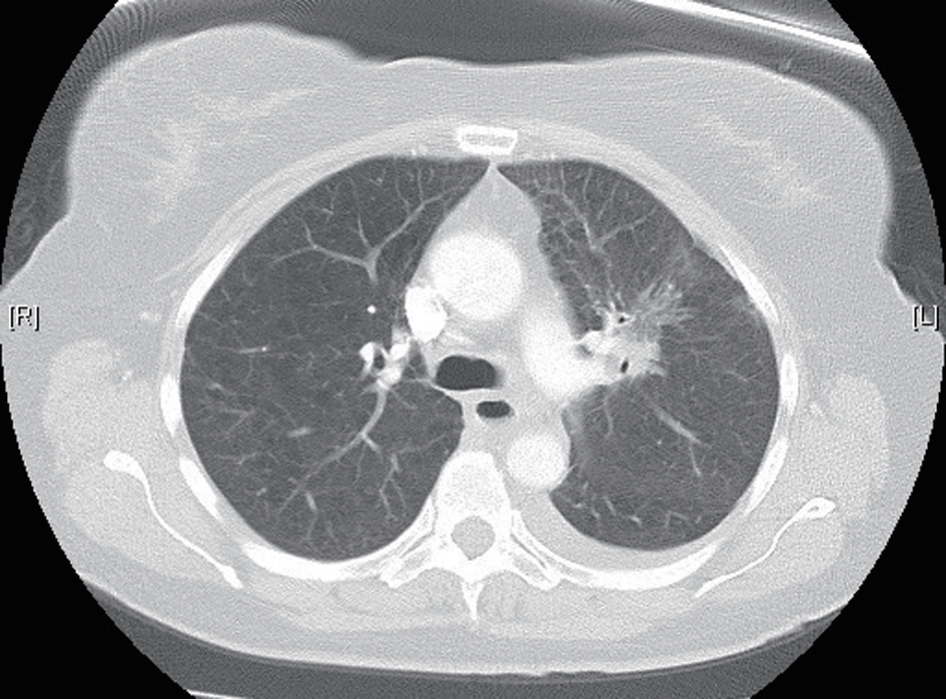
High-resolution lung computed tomography before pemetrexed administration.

A 61-year-old female was diagnosed with stage IV (T2a, N2, M1b) NSCLC in June 2012. Positron emission tomography (PET) scan showed evidence of oligometastatic disease involving the right adrenal gland. She subsequently had a laparoscopic resection of the right adrenal gland in June 2012; pathology results were consistent with a metastatic, poorly differentiated adenocarcinoma of lung origin. She began concurrent chemoradiotherapy in July 2012, with cisplatin and etoposide, and radiotherapy (60 Gy). Treatment was complete by August 2102. Restaging computed tomography (CT) scan showed regression of the primary tumor. There was no evidence of any interstitial lung disease (Fig. 1). In October 2012, 56 days following completion of radiotherapy, maintenance pemetrexed was commenced, 500 mg/m^2^ every 21 days, with supplemental vitamin B12 and folic acid.

**Figure 2:**
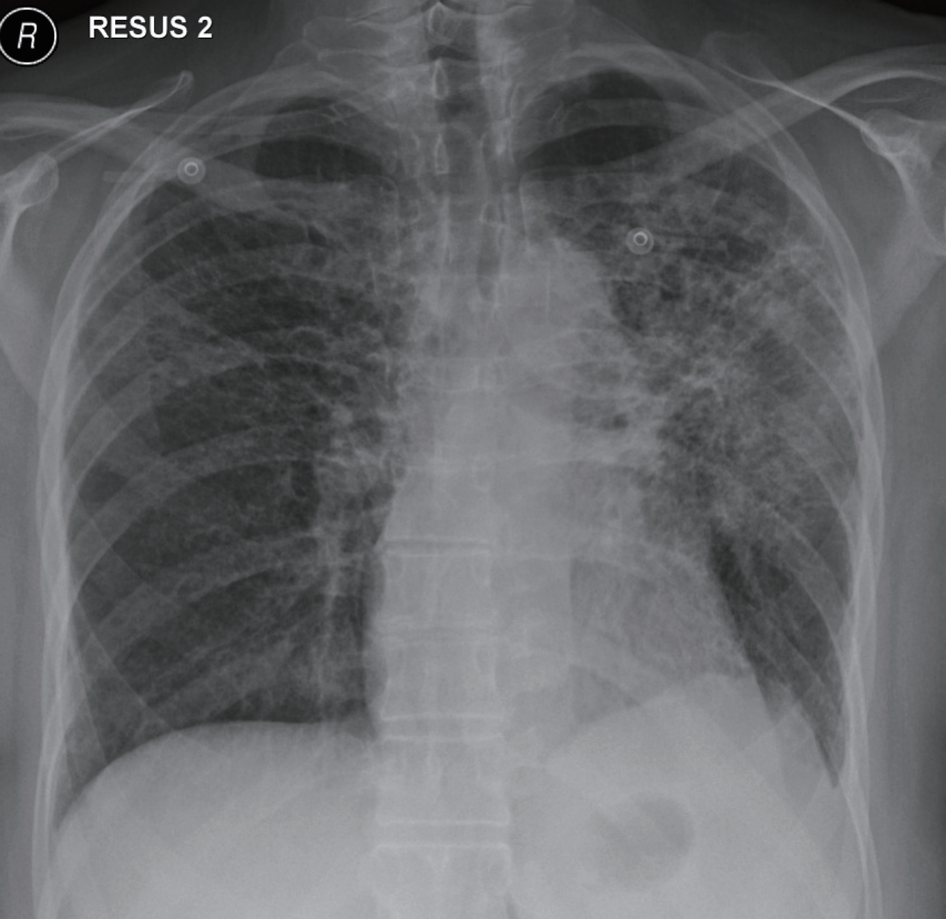
Chest X-ray on initial presentation, showing diffuse bilateral reticulonodular opacities in the upper lobes.

**Figure 3:**
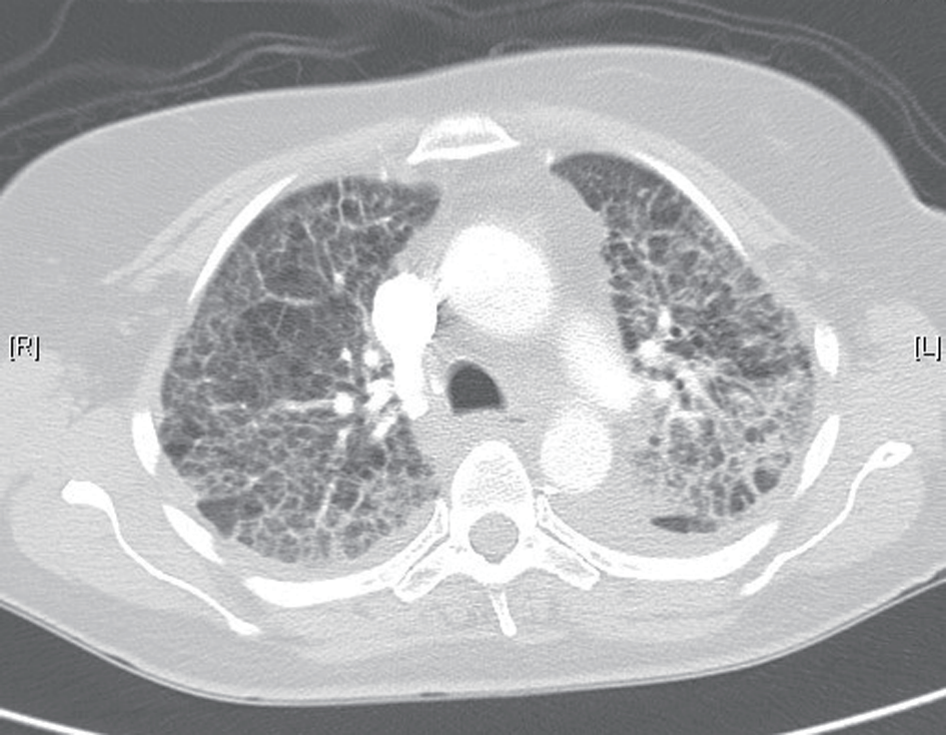
Lung computed tomography during acute illness, showing bilateral diffuse interstitial changes and ground-glass opacities.

In November 2012, 13 days after the second dose of pemetrexed, the patient presented to our tertiary referral center after developing fever, cough, lethargy and dyspnoea at rest. She had no chest pain or pedal edema. On initial examination, the patient was febrile (temperature 38 °C) and in visible respiratory distress with a respiratory rate of 30 breaths/min and oxygen saturation 78% on room air. An arterial blood gas analysis taken on 8 L/min of oxygen showed hypoxia with a respiratory alkalosis (pH 7.47, PO_2_ 73 mm Hg, and PCO_2_ 32 mm Hg). Chest X-ray showed diffuse reticulonodular opacities in the upper lobes bilaterally (Fig. 2). CT scan revealed widespread interstitial opacity with inter- and intra-lobular septal thickening, and fine nodularity throughout both lung fields (Fig. 3). In some regions the diffuse interstitial opacity gave a ground glass appearance. The radiological changes were diffuse, including areas outside of the previous radiotherapy field. There was no evidence of pulmonary embolus. Her white cell count was not elevated (4 × 10^9^/L; neutrophils 3.5 × 10^9^/L) and hemoglobin level was 101 g/L. Tests of renal and liver function were normal. C-reactive protein was 170 mg/L (normal < 8 mg/L). She was commenced on broad spectrum intravenous antibiotics and transferred to the intensive care unit (ICU) due to ongoing hypoxia and respiratory distress. In the ICU she was commenced on intravenous dexamethasone and placed on non-invasive ventilation. She improved rapidly over the next 3 - 4 days. Repeated blood cultures and induced sputum did not reveal evidence of a typical or atypical infective cause. Bronchoscopy was not performed as the patient was not fit for the procedure whilst acutely unwell. She improved rapidly with conservative management.

**Figure 4:**
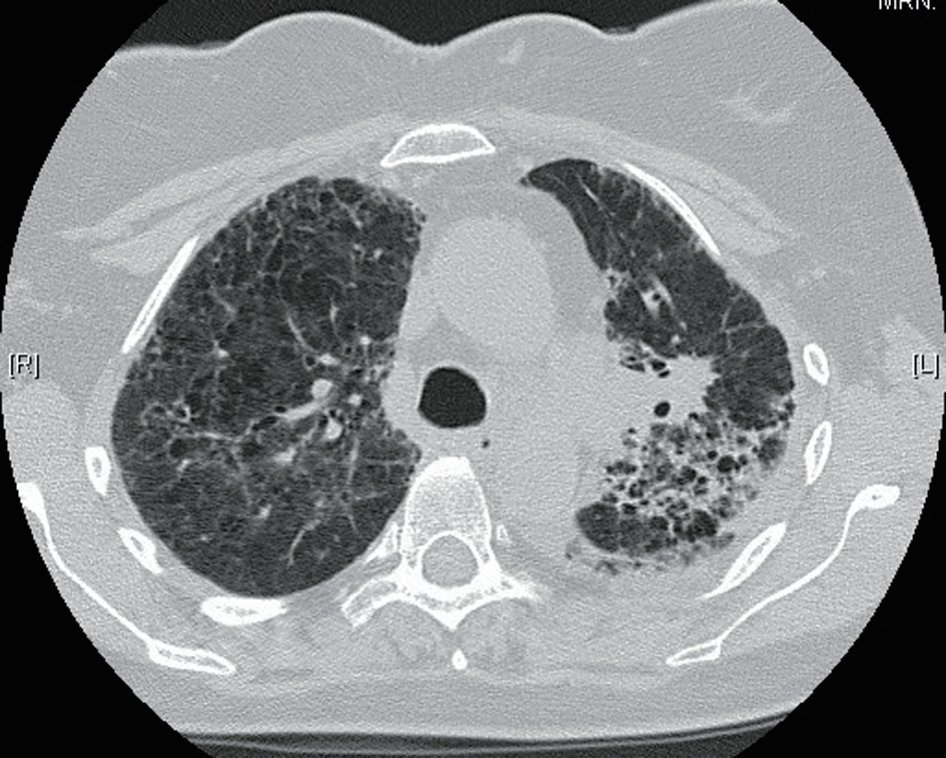
High-resolution lung computed tomography 1 month post-illness, showing interval resolution of interstitial changes.

**Figure 5:**
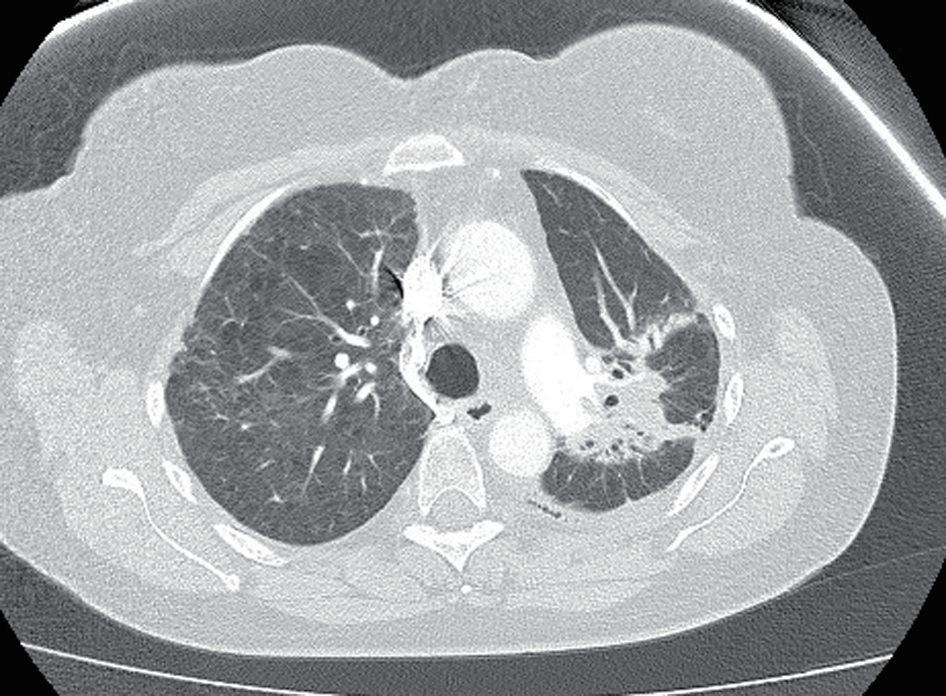
Further resolution on computed tomography, 4 months post-illness.

The patient returned home 10 days following hospital admission. She was not rechallenged with pemetrexed. High-resolution CT 1 month following her hospital admission showed interval resolution of the ground-glass attenuation, and complete resolution of the alveolar changes present during acute illness (Fig. 4). There was further resolution evident on repeat CT scanning 4 months post-illness (Fig. 5). She is currently on surveillance.

## Discussion

Chemotherapy-induced lung toxicity occurs in less than 10% of patients on cytotoxic agents; however, it can be severe, rapidly progressive and potentially fatal [8]. Methotrexate, bleomycin, gemcitabine and cyclophosphamide are the best-known causative agents. Interstitial pneumonitis is the most common presentation of chemotherapy-induced lung toxicity [8].

The use of pemetrexed as a chemotherapeutic agent is growing, given recent evidence supporting its use in patients with NSCLC and malignant mesothelioma. Acute lung injury was not reported as a side effect of pemetrexed from the phase III clinical trials leading to its widespread use for NSCLC [3, 7]. Since then, Ohe et al [9] reported eight cases of interstitial pneumonitis in patients treated with pemetrexed, although sufficient clinical data linking the two were lacking. Performing a comprehensive literature review, we identified eight case reports in the English literature linking pemetrexed to acute lung injury [10-15] (Table 1).

**Table 1:** Reported Cases of Pemetrexed-Induced Pneumonitis

Case	Number of cycles	Time of symptom onset after last dose	Prior radiation	BAL	Biopsy confirmation	Rechallenged	Outcome
Loriot et al 2009 [10]	5	3 days	No	Yes	No	No	Returned to pre-morbid status. Signs of pulmonary fibrosis on CT.
Kim et al 2010 [11]	4	“Shortly after”	No	No	Yes	No	“Slow improvement”.
Nagata et al 2010 [12]	1	4 weeks	No	No	No	No	Died 3 weeks after symptom onset.
Nagata et al 2010 [12]	1	3 weeks	No	No	No	No	Died 2 weeks after symptom onset.
Breuer and Nechushtan 2011 [13]	4	-	No	No	No	Yes	Pneumonitis recurred after rechallenge. Symptoms resolved in 2 weeks.
Hochstrasser et al 2012 [14]	2	1 week	Yes	No	No	Yes	Pemetrexed was continued. Patient died 5 days after next dose.
Hochstrasser et al 2012 [14]	2	-	No	Yes	No	No	Improved rapidly; symptoms resolved.
Kim et al 2013 [15]	1	3 weeks	No	Yes	Yes	No	Recovered; commenced erlotinib.
Waters et al 2013	2	13 days	Yes	No	No	No	Improved rapidly.

BAL: Bronchoalveolar lavage; CT: computed tomography; (-): information not available from case report.

There is no definitive diagnostic test for pemetrexed-induced interstitial pneumonitis. Diagnosis remains one of exclusion, based on clinical manifestations, radiographic abnormalities, bronchoalveolar lavage (BAL) analysis, lung histology when available, and resolution of symptoms after drug discontinuation. Bacterial and viral infection should be excluded. Lymphangitis carcinomatous and radiation pneumonitis should also be considered in the differential diagnosis. Definitive diagnosis remains a challenge for clinicians, as clinical and histopathological signs are non-specific. All eight patients from the previously reported case studies (Table 1) presented with acute onset dyspnoea and cough whilst on pemetrexed therapy for NSCLC or advanced mesothelioma. The majority of cases also presented with fever, although an infective source was not isolated in any of the cases.

It is arguable whether all eight reported cases can be conclusively linked to pemetrexed administration. An infective source was actively excluded in all cases, and imaging was consistent with interstitial pneumonitis. None of the eight patients had a previous history of interstitial lung disease. Only one of the eight case studies reported prior radiation therapy, concluding 76 days prior to onset of symptoms [14]. However, only two cases were supported with lung biopsy [11, 15], showing a picture of diffuse alveolar damage, alveolar wall thickening and mononuclear inflammatory cell infiltrate. These patterns are non-specific, seen in infections and other drug-induced lung injury, including that due to methotrexate [16]. BAL was performed in three of the eight cases [10, 14, 15]. This investigation is not specific for drug-induced pneumonitis, but may help to rule out infection and lymphangitic tumor. Lymphocytosis and an increase in the CD4/CD8 ratio has been reported from BAL in patients with methotrexate-induced pneumonitis [17].

In our case, we did not have the benefit of lung biopsy or BAL. However, the lack of infective organism isolated, and the strong response to steroids coupled with interval radiological resolution at 1 month, supports a drug-induced mechanism. Radiation-induced pneumonitis is a well-documented side effect of radiotherapy and was considered in our differential diagnosis. It usually conforms to the prior radiation field, although widespread pneumonitis outside the irradiated field has also been reported [18]. Onset is classically 4 - 6 weeks following irradiation [19]. Mean durations from treatment completion to clinical onset of 14.9 days [20] and 21 days [21] respectively have been reported. Our patient completed her course of radiotherapy 13 weeks (90 days) before the onset of pneumonitis, making a direct causal relationship less likely. However, radiation pneumonitis cannot be completely excluded. Radiation-recall pneumonitis, another differential diagnosis, is a poorly understood phenomenon, associated with multiple chemotherapeutic agents [22, 23]. Clinical onset can occur months to years after radiotherapy, although lung pathology usually conforms to the prior radiation field [24].

Methotrexate is known to cause interstitial pneumonitis [25], with most researchers suggesting a hypersensitivity mechanism resulting in lung injury [26, 27]. Usual presence of fever, eosinophilia and a mononuclear cell infiltrate of the lungs on biopsy support this mechanism [27]. The mechanism of pemetrexed-induced interstitial pneumonitis may also be a hypersensitivity reaction. CD4/CD8 ratios from BAL in two of the case studies support a hypersensitivity hypothesis [10, 14].

Another possible mechanism of pemetrexed-induced interstitial pneumonitis may be a toxic drug reaction resulting from accumulation within lung tissue, supported by the resolution of most cases after pemetrexed cessation. However, from the eight reported cases in the literature, clinical onset of pneumonitis does not appear to relate to the duration of pemetrexed therapy. The same holds true for cases of methotrexate-induced lung injury, where dose and duration of drug therapy do not correlate with onset or severity of lung injury [16].

From the eight reported cases of pemetrexed-induced pneumonitis, three patients (38%) died, with one following re-exposure to pemetrexed. The others (62%) showed signs of improvement following initial presentation, some of these with rapid return to pre-morbid function following the cessation of pemetrexed and administration of steroids.

We did not rechallenge our patient with pemetrexed after her initial presentation, and believe it may be unwise to do so. Two of the previously reported cases reintroduced pemetrexed; one case resulted in a fatal outcome [14], and the other had symptom recurrence which then abated on further pemetrexed cessation [13]. Although there is some evidence to suggest the reintroduction of methotrexate after methotrexate-induced lung injury is safe in the majority of cases [16], we believe the risks of rechallenging with pemetrexed outweigh the benefits, given our current knowledge.

### Conclusion

Interstitial pneumonitis appears to be a rare side effect of pemetrexed therapy. Diagnosis should be suspected when there is a temporal association and other causes have been excluded. Treatment is conservative with steroids and temporary ventilatory support may be required. Radiotherapy may predispose patients to this complication; further clinical studies are required. Rechallenging with pemetrexed after the initial presentation is not advised and may have fatal consequences.

## Author Note

The study was performed at Flinders Medical Centre and Flinders University School of Medicine, Bedford Park, Adelaide, South Australia 5042.
